# Use of the KT-MCC strategy to improve the quality of decision making for multidisciplinary cancer conferences: a pilot study

**DOI:** 10.1186/s12913-020-05143-3

**Published:** 2020-06-24

**Authors:** Christine Fahim, Meghan M. McConnell, Frances C. Wright, Ranil R. Sonnadara, Marko Simunovic

**Affiliations:** 1 Department of Health Research Methods, Evidence and Impact, McMaster University, Hamilton, ON Canada; 2 Bloomberg School of Public Health, Johns Hopkins University, Hampton House, Room 663, 624 N Broadway, Baltimore, MD 21205 USA; 3 Department of Innovation in Medical Education, University of Ottawa, RGN 2156, 451 Smyth Rd, Ottawa, ON K1H 8M5 Canada; 4 Department of Surgery, University of Toronto, Toronto, ON Canada; 5 Sunnybrook Health Sciences Centre, Room T2 057, 2075 Bayview Ave, Toronto, ON M4N 3M5 Canada; 6 Department of Surgery, McMaster University, Hamilton, ON Canada

**Keywords:** Integrated knowledge translation, Theoretical domains framework, Behaviour change wheel, Knowledge-to-action cycle, Multidisciplinary Cancer conference, Multidisciplinary decision making, Cancer

## Abstract

**Background:**

Multidisciplinary Cancer Conferences (MCCs) are increasingly used to guide treatment decisions for patients with cancer, though numerous barriers to optimal MCC decision-making quality have been identified. We aimed to improve the quality of MCC decision making through the use of an implementation bundle titled the KT-MCC Strategy. The Strategy included use of discussion tools (standard case intake tool and a synoptic discussion tool), workshops, MCC team and chair training, and audit and feedback. Implementation strategies were selected using a theoretically-rooted and integrated KT approach, meaning members of the target population (MCC participants) assisted with the design and implementation of the intervention and strategies. We evaluated implementation quality of the KT-MCC Strategy and initial signals of impact on decision making quality.

**Methods:**

This was a before-and-after study design among 4 MCC teams. Baseline data (before-phase) were collected for a period of 2 months to assess the quality of MCC decision making. Study teams selected the intervention strategies they wished to engage with. Post-intervention data (after-phase) were collected for 4 months. Implementation quality outcomes included reach, adherence/fidelity and adaptation. We also evaluated feasibility of data management. Decision making quality was evaluated on a per-case and per-round level using the MTB-MODe and MDT-OARS tools, respectively.

**Results:**

There were a total of 149 cases and 23 MCCs observed in the before phase and 260 cases and 35 MCCs observed in the after phase. Teams implemented 3/5 strategies; adherence to selected strategies varied by MCC team. The per-round quality of MCCs improved by 11% (41.0 to 47.3, *p* = < 0.0001). The quality of per-case decision-making did not improve significantly (32.3 to 32.6, *p* = 0.781).

**Conclusion:**

While per round MCC decision making quality improved significantly, per-case decision-making quality did not. We posit that the limited improvements on decision making quality may be attributed to implementation quality gaps, including a lack of uptake of and adherence to theoretically-identified implementation strategies. Our findings highlight the importance of evaluating implementation quality and processes, iterative testing, and engagement of key gatekeepers in the implementation process.

## Background

Multidisciplinary Cancer Conferences (MCCs) are regular meetings to discuss treatment plans for patients with cancer. Usual MCC participants include surgeons, medical and radiation oncologists, pathologists and radiologists. MCCs can increase rates of optimal staging, decrease time from diagnosis to treatment, increase use of neoadjuvant therapy, improve collaboration among team members, decrease patient wait times, and in some cases, improve patient outcomes [1–5]. MCCs are prevalent worldwide and recent evidence suggests most cancer cases would benefit from a multidisciplinary review [6].

Cancer Care Ontario (CCO), the agency responsible for improving the care received by Ontario patients with cancer, mandated in year 2010 the use of disease-specific MCCs (e.g., lung, hepatobiliary, gastrointestinal) at hospitals that treat a high-volume of patients with respective diagnoses (e.g., breast MCC if site treats > 35 unique patients with breast cancer) [7]. Prior to this mandate, CCO released a MCC best practice standards document and developed a scorecard to evaluate MCC quality [7]. This scorecard evaluates frequency of MCCs at eligible Ontario hospitals and attendance by seminal specialties. Recent data show that 85% of Ontario hospitals have achieved concordance with CCO standards [8].

While the CCO initiative has greatly increased the use of MCCs in Ontario hospitals, there has been no evaluation of the quality of decision making resulting from such MCCs. Our own group, using qualitative interviews and focus groups with Ontario MCC participants, found numerous barriers to optimal MCC decision making. Gaps in quality have also been measured in Ontario and elsewhere [9]. Despite identified gaps, there are few strategies to improve MCC processes [10–12].

We aimed to develop a knowledge translation (KT) [13, 14] strategy to optimize MCC decision making that would be relevant to an Ontario context. We used KT theories, models and frameworks, and an integrated KT (iKT) approach with MCC stakeholders to identify barriers to optimal decision making and potential interventions to overcome these barriers. Experts recommend the use of theory to inform the design and implementation of quality improvement interventions [15]. iKT approaches demand the inclusion of targeted stakeholders in the design, implementation and evaluation of a KT intervention, and are thus believed to better engage target populations and improve the chances of intervention success [13, 14].

Prior to a randomized trial to measure effectiveness of the KT-MCC Strategy, we aimed to evaluate implementation outcomes of the KT-MCC Strategy and initial signals of impact on quality of MCC decision making. The research questions on quality of implementation (as defined by Durlak and Dupre) [16], included: *What was the reach of the KT-MCC Strategy (RQ1), What adaptations to the Strategy were made by MCC teams (RQ2), What is the adherence/fidelity of implementation of selected strategies (RQ3)*. Other questions included *What is the feasibility of management* (i.e., data collection and management issues) as defined by Thabane et al’s [17] guidance for pilot studies (*RQ4*); *What is the impact on per case decision making (RQ5) and per round decision making (RQ6) quality; What are the factors associated with a high quality per case decision (RQ7); What qualitative themes regarding implementation of the KT-MCC Strategy were identified? (RQ8).*

## Methods

### Study design, setting and participants

This was a prospective, before-and-after pilot study. Four Ontario MCC groups participated in the pilot. Three of the four participating MCC teams were located at a single, academic hospital. One MCC team was located at a community hospital. MCC participants included surgeons, medical and radiation oncologists, radiologists and pathologists. Some rounds were attended by nurses, pharmacists and trainees.

### Development of the KT-MCC Strategy

Methods to develop the KT-MCC Strategy are provided in detail elsewhere [18]. We used the Knowledge-to-Action (KTA) cycle as our conceptual model. In our previous work, we describe our process of *Identifying the Problem*, *Adapting Knowledge to the Local Context*, and *Assessing Barriers and Facilitators to Knowledge Use*, to develop our intervention (titled the KT-MCC Strategy) [19]. Here, we describe the *Selection, Tailoring and Implementation* phase of implementation, in keeping with the KTA model [19].

We developed the KT-MCC Strategy using theory and an iKT approach. Interventions that are not theoretically-rooted may overlook important mediators of behaviour, potentially resulting in less effective interventions [15]. In addition, use of a theoretical framework provides testable models that can explain the success or failure of the intervention of interest [15, 20–23]. Key informant interviews, guided by the Theoretical Domains Framework (TDF), were used to identify barriers and facilitators to optimal decision making [24, 25]. Identified domains of behaviour change were mapped to the COM-B Behavioural Change Wheel to identify corresponding intervention functions, which in turn were used to select intervention strategies to inform development of the KT-MCC Strategy [26]. Focus group informants confirmed the face validity of the key informant data and confirmed the acceptability of the proposed strategies. To facilitate iKT, MCC participants (the target population) were involved in the design and implementation of the intervention. The KT-MCC Strategy, derived through the theoretical mapping, included a workshop to develop local consensus processes; MCC team training; MCC chair training; use of a standard intake tool; use of a synoptic discussion tool; and, audit and feedback, delivered monthly to the MCC chair. A detailed description of the KT-MCC Strategy intervention components is provided in Appendix 1.

### iKT approach

We used an iKT approach to develop the KT-MCC Strategy, meaning members of the target population (i.e., MCC attendees) informed the development and implementation of the intervention. Specifically, MCC chairs were identified as key stakeholders, and often acted as the gatekeepers to individual MCC rounds. We therefore involved MCC chairs in the implementation and evaluation of the KT-MCC Strategy.

### Outcomes

Implementation outcomes were classified according to Durlak and Dupre’s [16] process outcomes for implementation research and included:

- Reach: The number of sites approached to participate versus the number that agreed to participate
- Adaptation: Reported adaptations to KT-MCC strategies, made by the individual MCC teams
- Adherence/Fidelity: A priori, we defined acceptable adherence as implementation at a minimum rate of 80% (e.g., if the synoptic checklist was selected, it should be used for at least 80% of cases). Fidelity (i.e., how closely the implementation reflected the original Strategy components) was evaluated through observation and by using administrative data (i.e., submitted standard intake forms).

Feasibility of management (as defined by Thabane et al.) [17] was defined as the research team’s ability to gain access to MCCs for observation and collect relevant study data (evaluated using study documents and observations). We also report the time spent per case and per round as a measure of feasibility. Additionally, we evaluated initial signals of impact on MCC quality of decision making. This was evaluated at the case level (i.e., quality of decision making for an individual case of a patient with cancer) and at the round level (i.e., quality of each MCC round). We determined whether certain factors identified through the literature as important to MCC decision making (e.g., most responsible physician presents their own case at rounds) were correlated with improved decision making quality. Finally, we report the qualitative observations gathered through field notes during the implementation process.

### Data collection

We used the Multidisciplinary Tumor Board Metric of Decision Making (MTB-MODe) to assess *per case* MCC decision making quality. The tool was developed by Lamb et al. to evaluate the quality of decision making in urology and colorectal MCCs in the United Kingdom [9, 11, 27, 28]. Previous work by our group demonstrates that the MTB-MODe can be used in a North American context to reliably distinguish high versus low quality MCC decision making, and can be successfully implemented using a single rater [29]. The MTB-MODe evaluates the quality of per case decision making quality on two domains: quality of information presented (six items) and quality of teamworking (five items), anchored on a Likert scale of 1–5 (where 1 indicates low quality and 5 indicates high quality) [27, 28]. Non-attendance by a core specialty was recorded as N/A on the MTB-MODe tool (as opposed to a score of 1) to allow the research team to distinguish between non-attendance and low teamworking contribution. MTB-MODe items were summed for a minimum score of 6 and a maximum score of 55 (maximum scores of 30 and 25 for quality of information presented and quality of teamworking, respectively).

We used the Multidisciplinary Team Observational Assessment Rating Scale (MDT-OARS) to evaluate per round MCC decision making quality [30–32]. The tool identifies 17 aspects of multidisciplinary teamworking that impact MCC decision making (e.g., team characteristics, infrastructure, meeting organization and logistics, and patient-centered decision-making). The tool is based on *The Characteristics of an Effective Multidisciplinary Team* guideline published by the English National Cancer Action Team [32]. Developers of the tool report its strong internal consistency and inter-rater reliability [30, 31]. The tool has been successfully used to evaluate the quality of decision making for a colorectal MCC in the UK [33]. All items are summed to report a single quality of decision making per round score. The maximum score that could be awarded to an MCC round was 57 and the lowest was 15.

Baseline data (before phase) were collected for a period of 2 months. Post-intervention data (after phase) were collected for 4 months. The decision to extend the post-intervention period was to ensure that enough time was allocated for teams to adapt and implement the strategies as needed, which is in keeping with guidance from the Knowledge-to-Action model [19]. A single, trained researcher evaluated per case and per round quality and took field notes regarding implementation processes.

### Data analyses

#### Implementation outcomes and feasibility

Measures were calculated (e.g., using administrative data and intake forms) or described following direct observation, as appropriate. A single researcher trained in qualitative research analyzed the field notes to identify emergent themes.

#### Decision-making outcomes

Univariate analyses using independent samples student’s t-tests (two-tailed with a significance level of 0.05) were used to compare in the before versus after periods the quality of decision making scores (MTB-MODe - score per case, MDT-OARS - score per round) for each MCC team and across MCC teams. Descriptive statistics were provided for time spent per case on case history and overall discussion, and number of cases presented per round. We also conducted multivariate analyses using a generalized linear model (GLM) to determine whether the KT-MCC Strategy had a significant impact on per case MTB-MODe scores. Unlike a traditional ANOVA, the GLM was selected to account for data that violates the homoscedasticity assumption. Each GLM model defined the dependent variable as the MTB-MODe score. The independent factors were: the before/after label (1 = pre-intervention scores; 2 = post-intervention score) and the MCC team ID (1–4). We used a multiple linear regression model to determine if the presence of the following factors correlated with increased MTB-MODe scores: clear clinical question articulated at time of case presentation, case submitted using standard intake form, final treatment plan articulated by MCC chair. Statistical data were analyzed using SPSS 23 software. This study was approved by the respective Research Ethics Boards of participating sites.

## Results

### Description of MCC rounds

Rounds took place on a weekly or biweekly basis. All MCCs were intended to last 60–80 min; however, MCC rounds lasted for an average of 40.7 min (range: 8–93 min; median: 36.5 min). Over the course of this study, technological improvements were made to the academic hospital hosting MCCs 1, 2, and 3 (e.g., provision of a microscope to view pathology slides during rounds) and an information expert was available at the start of each of these rounds to ensure MCC teleconferencing ran smoothly. MCCs were primarily led by chairs who facilitated a discussion among staff physicians; residents did not often participate in the MCC discussion.

Baseline (before period) and post-implementation (after period) data were collected from January–March 2017 and June–September 2017, respectively. There were a total of 149 cases and 23 MCCs observed in the before phase and 260 cases and 35 MCCs observed in the after phase.

### Process and implementation outcomes

#### Reach

Seven MCC teams (four teams at single academic institution; three teams within three different community hospitals) were approached to participate in the KT-MCC Strategy and all agreed. All invited teams presented a minimum of six cases per round and met on a weekly or biweekly basis. Time restraints with one academic team, and delayed ethics board approvals with two community hospital teams precluded participation in the pilot study.

#### Adaptation and Fidelity

We met with each MCC chair individually to review baseline quality scores and present the potential KT-MCC Strategy intervention components. Chairs were then responsible for next steps (e.g., selecting interventions for implementation) and methods of next steps (e.g., unilateral implementation decisions made by the chair or decisions made by the entire MCC team). The MCC chairs of the four teams that participated solicited the team’s opinions on the acceptability of the interventions, yet ultimately determined which interventions were selected for implementation. All chairs were agreeable to implement workshops to develop local consensus processes, the standard intake form and synoptic discussion checklist, and, audit and feedback. It took teams 3–5 weeks to hold the workshops, implement local consensus processes and tailor the standard intake forms. Teams adapted the checklist and discussion form to suit their needs (e.g., checkbox enabled feature on form added for MCC4). None of the chairs selected chair or team training for implementation, despite some feedback from MCC teams in support of the training interventions.

Each team developed a set of local consensus processes outlining MCC processes and data items to be tracked, via a workshop (see Table 1). Adherence to selected local consensus processes varied by team (see Table 2). There was lack of attendance by seminal specialties in up to 50% of cases. Adherence to the defined weekly maximum number of cases ranged from 20 to 100%.

**Table 1:** Adoption of KT-MCC Strategy intervention components & local consensus processes, by MCC team

KT-MCC Strategy Component	MCC Team 1	MCC Team 2	MCC Team 3	MCC Team 4
**Local Consensus Processes**	-Deadline for case submission -Maximum number of cases defined -Maximum discussion time per case defined -Requirement to attend MCCs on time -Requirement for MRP to attend (or send surrogate) in order to present case	-Deadline for case submission -Maximum number of cases defined -Deadline to submit imaging -Requirement for MRP to attend (or send surrogate) in order to present case -Types of cases to be discussed defined	-Deadline for case submission -Maximum number of cases defined -Maximum discussion time per case defined -Requirement to attend MCCs on time	-Deadline for case submission -Deadline to submit imaging
**Standard Intake Form; Synoptic Discussion Checklist**	-Agreed to use synoptic checklist -Clear clinical question and original treatment plan required on intake form -Agreed to collect data regarding rate of decision change following discussion -Treatment plan to be articulated by chair -Chair agreed to invite members of each specialist group to participate in discussion	-Agreed to use synoptic checklist -Chair to control discussion to a moderate extent -Treatment plan to be articulated	-Agreed to use synoptic checklist -Clear clinical question and original treatment plan required on intake form -Agreed to collect data regarding rate of decision change following discussion -Treatment plan to be articulated by chair and disseminated back to group	-Agreed to use synoptic checklist -Clear clinical question and original treatment plan required on intake form -Agreed to collect data regarding rate of decision change following discussion -Treatment plan to be articulated by the chair -Chair agreed to invite members of each specialist group to participate in discussion
**Chair Training**	–	–	–	–
**Team Training**	–	–	–	–
**Audit and Feedback**	A&F for -Rate of decision change -Time spent per case -Cases discussed per round -Quality of information -Quality of teamworking	A&F for -Rate of decision change -Time spent per case -Cases discussed per round -Quality of information -Quality of teamworking	A&F for -Rate of decision change -Time spent per case -Cases discussed per round -Quality of information -Quality of teamworking	A&F for -Rate of decision change -Time spent per case -Cases discussed per round -Quality of information -Quality of teamworking


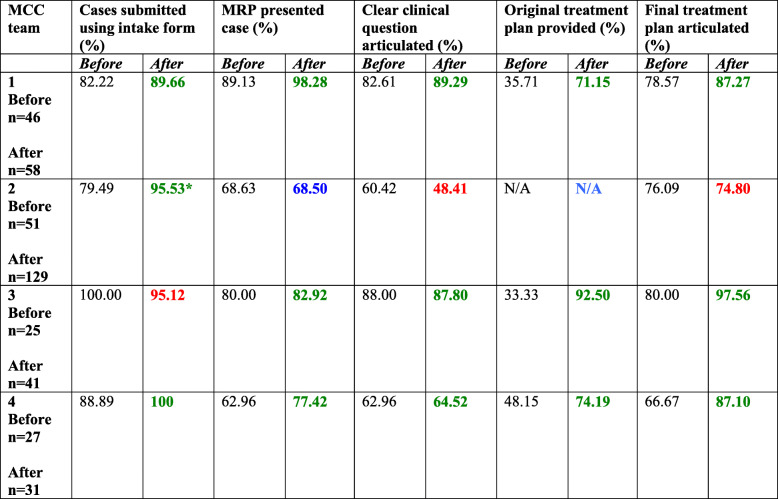

**Table 2:**
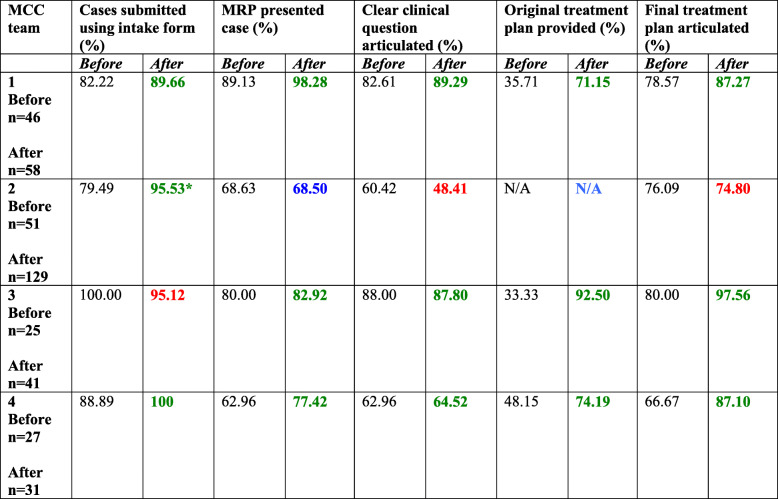
Fidelity to selected interventions before/after KT-MCC Strategy

*KT-MCC Strategy standard intake form not used – team reverted to original intake form

Red: Denotes regression in compliance; Green: Denotes improvement in compliance; Blue: Denotes no change in compliance

Three of the four teams used the standard intake form for 100% of discussed cases, and one team used the form for 25% of cases. Completion of items on the standard intake form ranged from 48 to 100% (see Table 2). None of the teams were compliant with the synoptic discussion checklist – we did not observe any formal implementation of the tool to guide MCC discussion or decision-making.

We observed formal dissemination of the audit and feedback data by two chairs (observed once per team during the study period). We did not observe any other dissemination of the audit and feedback data to study teams by MCC chairs.

#### Feasibility

We did not encounter any challenges with the data collection process [17]. The KT-MCC Strategy did not impact time spent per case discussion. Time spent per case was 6 min at baseline and decreased by only 20 s in post-intervention mean (before = 359.5 s, SD 204.6 s; after = 339.9 s, SD 184.3 s, *p* = 0.35). The time spent per case discussion and time spent on case history had a significant inverse correlation with the order in which the case was presented (*p* = 0.000, *p* = 0.000, respectively), meaning cases presented at the start of each MCC received a longer discussion.

### Decision making outcomes

#### Univariate analyses – impact on per case decision making

MTB-MODe scores were available for 409 cases from 58 rounds (see Table 3). Aggregate scores did not improve with use of the KT-MCC Strategy (see Table 4). In the pre- and post-intervention periods, the mean scores for quality of information provided were 17.1 and 17.8 (*p* = 0.255), respectively, and for quality of teamworking were 15.2 and 15.9 (*p* = 0.198), respectively (see Table 4). The pre and post composite scores for per case decision making quality were 32.3 to 32.6 (*p* = 0.781), respectively. Scores for MCC 1 improved significantly for both quality of information presented (17.60 to 19.26) and quality of teamworking (15.60 to 18.26) (*p* = 0.002, *p* = 0.044, respectively). Scores did not improve significantly for MCCs 3 and 4. Finally, quality of information and teamworking scores decreased for MCC 2 (16.49 to 15.19, *p* = 0.185; 16.26 to 14.01, *p* = 0.014, respectively) (see Table 4).

**Table 3:**
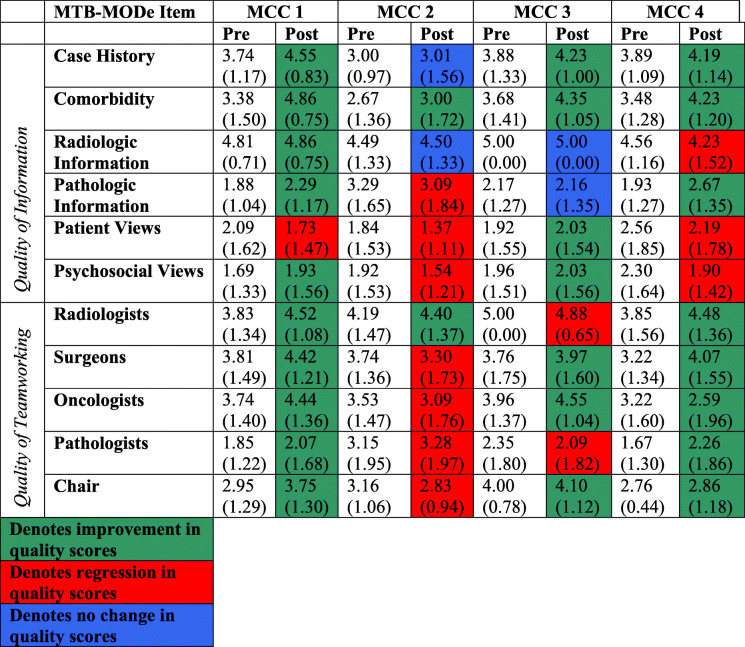
Descriptive statistics for quality of MCC decision making per case, by team

**Table 4:** Effect of KT-MCC Strategy on Per Case MCC Decision Making Quality

	Scores for MCC1–4			MCC 1			MCC 2			MCC 3			MCC 4		
	Pre (***n*** = 149)	Post (***n*** = 260)	***p***	Pre (***n*** = 46)	Post (***n*** = 58)	***p***	Pre (***n*** = 51)	Post (***n*** = 129)	***p***	Pre (***n*** = 25)	Post (***n*** = 41)	***p***	Pre (***n*** = 27)	Post (***n*** = 31)	***p***
**Composite Score for Decision Making Quality**	32.30 (9.39)	32.59 (10.77)	0.781	33.19 (6.42)	37.77 (7.83)	0.002*	32.75 (9.47)	29.20 (11.61)	0.036*	34.52 (7.79)	34.90 (8.77)	0.854	32.82 (6.40)	35.13 (7.42)	0.207
**Quality of Information**	17.10 (5.63)	17.76 (5.48)	0.255	17.60 (4.62)	19.26 (4.63)	0.044*	16.49 (5.57)	15.19 (6.64)	0.185	18.52 (4.45)	19.20 (5.22)	0.578	18.70 (4.27)	19.32 (4.57)	0.596
**Quality of Teamworking**	15.20 (5.03)	15.87 (4.86)	0.198	15.60 (3.59)	18.26 (4.63)	0.002*	16.26 (5.31)	14.01 (5.81)	0.014*	16.00 (4.13)	15.71 (4.40)	0.786	14.11 (3.71)	15.81 (4.50)	0.122

Data presented in means (SD)

*Denotes a significant *p* value

#### Univariate analyses – impact on per round decision making

The summary MDT-OARS scores of decision making quality per round increased significantly from 40.96 (SD 4.46) in the pre- to 47.3 (SD 4.40) in the post-intervention period (*p* = < 0.0001) (see Table 5). Mean scores for 13 of the 15 items on the MDT-OARS improved following the KT-MCC Strategy, with the exceptions of *personal development* (i.e., observable communication of research evidence and/or instances of learning) and *availability of patient notes*. Less tension and conflict following the KT-MCC Strategy implementation was observed (− 0.41 pre-intervention to − 0.08 post-intervention).

**Table 5:** Effect of KT-MCC Strategy on Per Round MCC Decision Making Quality

	Attendance	Leadership	Inclusion of team members	Team Sociability	Mutual Respect	Personal Development	Meeting Venue	Technology & Equipment	Agenda	Prioritization of Case presentation	Availability of Patient Notes	Case Presentation	Patient Centered Care	Clarity of treatment plans	Presence of tension/conflict^a^	Summary Score of Overall MCC Quality	*p*-value for summary MCC quality score
**Pre**	3.14 (1.17)	2.41 (1.05)	3.14 (0.83)	3.59 (0.59)	3.23 (0.92)	1.50 (0.51)	3.77 (0.53)	5.00 (0.00)	2.62 (0.67)	3.55 (0.80)	4.00 (0.00)	2.16 (0.61)	1.32 (0.57)	2.17 (1.13)	−0.41 (0.96)	**40.96 (4.46)**	**< 0.0001**
**Post**	3.15 (1.25)	2.75 (1.15)	3.60 (0.84)	3.73 (0.64)	3.68 (0.69)	1.46 (0.60)	4.00 (0.00)	7.25 (1.32)	2.79 (0.47)	3.85 (0.48)	3.98 (0.16)	2.65 (0.59)	1.45 (0.75)	3.10 (0.76)	−0.08 (0.27)	**47.30 (4.40)**	
				**MCC 1**				**MCC 2**				**MCC 3**			**MCC 4**		
**Pre-Intervention MCC Quality Score**				38.92 (4.20)				43.17 (4.62)				41.67 (5.16)			39.63 (2.93)		
**Post-Intervention MCC Quality Score**				49.04 (2.23)				43.68 (5.07)				50.35 (2.89)			45.64 (3.54)		
***p*-value**				0.001				0.836				0.007			0.017		

Data presented in means (standard deviation)

^a^Evaluated on a negative scale (lower scores demonstrate greater levels of tension/conflict)

#### Factors correlated with decision making quality

Factors positively correlated with increased scores for per case MCC decision making quality included presentation of the case by the most responsible physician, submission of the case by the MCC deadline, provision of an original treatment plan by the most responsible physician, and, articulation of the final treatment plan (*p* = < 0.0001, *p* = 0.001 *p* = 0.006, and *p* = < 0.0001, respectively). Articulation of a clear clinical question by the most responsible physician prior to case discussion was not significantly correlated with increased decision making quality (*p* = 0.223) (see Appendix 2).

#### Observations and field notes

While chairs were ultimately responsible for running the MCC rounds, we observed non-chair opinion leaders intervening to ensure compliance with local consensus processes. For instance, these MCC participants consistently prompted their colleagues to articulate a clear clinical question to facilitate the case discussion. Teams without an engaged chair or the presence of such opinion leaders demonstrated observably lower quality of MCC decision making (e.g., MCC 2) compared to those that did (e.g., MCC 3). Some MCC coordinators also played a role in ensuring adherence to MCC processes – for instance, the coordinator for MCC 1 did not accept a case for presentation unless the standard intake form was completed in full. Residents were not active participants in rounds or the MCC discussion, although on few occasions they presented the case history on behalf of staff.

## Discussion

A review of 500 studies confirmed that good implementation quality is correlated with improved outcomes [16]. Studies that iteratively test for implementation problems demonstrate mean effect sizes 2–3 times greater than studies that do not evaluate implementation quality [16]. We aimed to pilot the KT-MCC Strategy to determine implementation quality prior to testing the strategy in a randomized trial to determine its impact on decision making quality.

While data management was feasible, this study identified a number of implementation gaps that must be addressed prior to further testing. First, delays in ethics approvals precluded the participation of 3/7 interested MCC teams, highlighting the amount of time required to effectively recruit participant sites to a future trial. Second, a theoretical mapping of identified barriers and facilitators to MCC decision making to corresponding intervention strategies revealed strategies that might improve MCC quality. These strategies composed the KT-MCC Strategy and included workshops to develop local consensus processes, use of a standard intake form, a synoptic discussion checklist, audit and feedback, and chair and team training. We used an iKT approach to select strategies for implementation; MCC chairs ultimately did not select team or chair training for implementation. This may explain why 2/4 MCC teams did not demonstrate significant improvements in teamworking scores on the MTB-MODe. Further, adherence with selected strategies was observed for some, but not all, of the selected intervention components – for instance, teams did not formally utilize the synoptic discussion checklist to guide case discussion. Additional research to identify more acceptable strategies to improve MCC teamworking and discussion is needed. Finally, we did not observe any improvements in MCC efficiency (e.g., time spent per case), however, trends did suggest an inverse correlation of time spent per case and order of case presentation. It is possible that complex cases were prioritized and discussed at the beginning of each round, which would explain this trend. Analysis of case type using tools such as Soukup et al.’s MeDIC tool [34] to evaluate case discussion complexity may provide additional insights.

We hypothesize that improved engagement of MCC chairs, who served as gatekeepers to MCC processes, and MCC opinion-leaders may improve adherence to selected strategies and subsequent implementation quality. Resident involvement in MCC discussions has also been reported to improve the quality of information presented [9]. Alternative models to MCCs that emphasize the role of these stakeholders should be considered.

We posit the KT-MCC Strategy led to changes in MCC processes (e.g., minimum required clinical information submitted by the MCC deadline using the intake form) and discussions (e.g., final treatment plan articulated for each case), which may explain the 11% improvement in quality per round. The availability of technical equipment (e.g., a microscope) and support may have also improved MTD-OARS scores. While improvements in MTB-MODe scores were not statistically significant, we identified a number of factors that correlated with improved decision making quality including presentation of the case by the most responsible physician, provision of an original treatment plan by the most responsible physician and submission of the case by a defined deadline.

There are few other examples in the literature that have implemented an intervention to improve MCC discussion quality. Lamb et al. implemented a multi-pronged strategy that included a synoptic reporting form, team training and written guidance in a urology MCC at a single hospital in the United Kingdom and evaluated impact using the MTB-MODe [9, 11, 27, 28]. The team noted a 9% and 5% significant absolute improvement in the quality of teamworking and information presented, respectively [9]. Similarly, Soukup et al. used a theoretically-rooted intervention of audit and feedback, short breaks, change of room layout and appointing a meeting chair to improve MTB-MODe scores for a breast cancer MCC in the UK [35]. While no improvements to quality of information were observed, teamworking scores improved by an average of 2 points on the scale (17.66 to 19.85) [35].

It is possible that we observed limited improvements to decision making quality in this study because of high baseline scores. For instance, the baseline mean score for quality of teamworking in our study was 61% compared to reported baseline MTB-MODe scores in the literature of 33–53% [11, 34]. KT literature dictates that quality improvement interventions are most effective when implemented among teams of lower quality, meaning teams with lower baseline scores are more likely to demonstrate a larger absolute intervention effect. It is also possible that our implementation period was not long enough to identify significant differences to implementation quality (e.g., 4 month implementation period versus Lamb et al.’s 16 months) [11]. Delivery of the KT-MCC Strategy over a prolonged time period might improve impact by allowing for additional iterations of adaptation.

Finally, our study was not without limitations. Time and resource constraints precluded multiple iterations of KT-MCC Strategy implementation among participating teams. In keeping with the premise of the Knowledge-to-Action cycle, we posit that additional, tailored interventions of the KT-MCC Strategy would result in improved Strategy adoption and fidelity [19]. Second, reliability tests were not performed for the MDT-OARS at our site due to logistical limitations (e.g., time availability of a second rater). However, iterations of the tool previously demonstrated strong inter-rater reliability (> 80%) and construct validity (*p* < 0.05) in a UK setting using multiple MCC disease sites [28, 36]. Third, it is possible that the presence of a rater during MCCs may have introduced a Hawthorne effect, however limited improvements in MCC quality mitigate this concern. Fourth, a single rater evaluated MCC decision making quality and may have been biased in their interpretations, particularly of teamworking. However previous work conducted by our study team demonstrates that the MTB-MODe can be effectively implemented by a single rater, given that individual raters did not contribute significantly to variance in scores in a generalizability study [29]. Fifth, this pilot study was evaluated among only four MCC teams working at two Ontario hospitals. Our results may not be generalizable to other settings. As well, we used a before and after study design. Observed improvements in per round decision making quality may not have been due to the KT-MCC Strategy but rather to other confounding factors. With regard to these latter two concerns, the main purpose of our study was to evaluate implementation outcomes for the KT-MCC Strategy. It is our intention to formally evaluate the effectiveness of the KT-MCC Strategy in a subsequent trial. Our findings and observations encourage such a study, however additional work to first improve implementation quality must take precedent.

## Conclusions

The KT-MCC Strategy is a theoretically-rooted intervention designed using an integrated KT process to improve the quality of MCC decision making. The KT-MCC Strategy was piloted for implementation outcomes and impact using a before and after design. We identified gaps in implementation quality that may have impacted improvements in MCC decision making scores. While we did not observe a significant improvement in per case decision making quality, the overall quality per MCC round improved. We posited the use of theory and an integrated KT approach would enhance adoption and feasibility of our KT intervention, however this was not fully actualized.

This work presents one of few studies that implemented and evaluated an intervention to improve multidisciplinary decision making quality in cancer care. We provide metrics for quality assessment and present simple MCC requirements that, if implemented, will likely improve decision making quality. Additional work to optimize engagement by MCC chairs and stakeholders and to promote the routine evaluation of MCC quality is warranted.

## Data Availability

The data generated and analysed during the current study are available from the corresponding author on reasonable request.

## Appendix 1

Workshops: Key informant data showed that MCC processes and goals differed by MCC team and disease-site. We therefore determined that a workshop to develop local consensus processes for each participating MCC team would be a necessary first step to implementing the KT-MCC Strategy [28, 29]. There were two main goals to the workshops: first, to review baseline data on each team’s MCC performance; and, second, to have participants develop local consensus processes for MCC functioning. The workshops facilitated adaptation of interventions using an integrated KT approach [14, 15]. Examples of local consensus processes include defining a maximum number of cases discussed per round or a deadline to submit cases to MCC coordinators.

Team Training: TDF interviews identified a lack of soft skills (e.g., effective communication and teamworking) leading to gaps in optimal MCC decision making quality. Team training was identified as an intervention to improve health professionals’ collaboration [26]. A team training expert with no previous relationship to the research team or MCC participants was identified to guide the training sessions. The expert was meant to guide MCC teams to identify “core values” required to build a strong foundation of teamworking and communication, and to provide the team with actionable strategies to engage in efficient and high quality decision making.

MCC Chair Training: Interview data reinforced that the MCC chair serves as a key arbiter of MCC processes. The chair usually ultimately determines how MCCs are organized, run, and recorded [1, 7]. Key informants correlated a lack of MCC leadership with cyclical case discussions, unclear final treatment plans, and an unequal degree of participation by seminal specialties. The purpose of the chair training session was to provide actionable solutions to MCC chairs on how to engage a MCC team, and how to help ensure efficient, comprehensive, and collegial MCC discussions. Chairs were to be taught how to effectively manage team conflict and to ensure all members of the MCC team are involved in the MCC decision making process [34, 35]. The same expert conducting the team training intervention was to lead the chair training.

Standard Intake Form: Lack of imaging at time of discussion, gaps in patient case history presentation, and lack of preparation by the presenting physician were identified as barriers to MCC decision making. A standard intake form was meant to improve the quality of information available at time of MCC discussion. The proposed intake form for the pilot study would require participants to submit details regarding patient demographics, clinical history, comorbidities, and whether a detailed review of pathologic or radiologic findings was required. Participants were to use the intake form to provide a clear clinical question, and an original treatment plan.

Synoptic Discussion Checklist: A synoptic discussion checklist developed by Lamb et al.’s research team was also disseminated to MCC teams [10, 30]. The purpose of the checklist was to prompt the chair to encourage input from seminal specialists and to ensure all pertinent patient information was considered. The checklist was also meant to prompt the MCC chair to articulate the final consensus treatment recommendations of the MCC team, to describe any objections to the treatment plan, and to indicate whether the case warranted further discussion at the subsequent MCC round.

Audit and Feedback: Audit and feedback involves measuring specific aspects of care provided by an individual or team, and providing this information back to the involved individuals - with or without comparator benchmarks - to encourage improvement [37]. Following input from MCC teams, we planned to audit and feedback relevant measures on MCC quality and efficiency. We planned to provide this feedback in writing to the MCC chairs, who would then be responsible for disseminating findings to their respective MCC teams.

## Appendix 2

Regression: Statistical Output

**Table:**

Model	R	R Square	Adjusted R Square	Std. Error of the Estimate
1 *N* = 364	.456	.208	.197	7.4839

**Table:**

Model	Unstandardized Coefficients		Sig.	95% Confidence Interval for B	
	B	Std. Error		Lower bound	Upper bound
(Constant)	54.488	2.355	.000	49.856	59.120
MRP presented their own case	−4.405	1.059	.000	−6.488	−2.323
MRP asked clear clinical question	−1.191	.975	.223	−3.110	.727
MRP provided original treatment plan	−2.342	.849	.006	−4.010	−.673
MRP submitted the case on time	−4.700	1.417	.001	−7.487	−1.914
Final, clear plan articulated	−4.372	1.034	.000	−6.405	−2.339

## Abbreviations

CCO: Cancer Care Ontario
COM-B: Capabilities, Opportunities, and Behaviour – Behaviour Change Wheel
KT: Knowledge Translation
KTA: Knowledge-to-Action Model
KT-MCC: Knowledge Translation-Multidisciplinary Cancer Conference Strategy (name of intervention)
MCC: Multidisciplinary Cancer Conference
MTB-MODe: Multidisciplinary Tumor Board Metric of Decision Making
MDT-OARS: Multidisciplinary Team Observational Assessment Rating Scale
TDF: Theoretical Domains Framework
